# The specialty of allergy and clinical immunology in Brazil

**DOI:** 10.3389/falgy.2022.933816

**Published:** 2022-07-18

**Authors:** Luane Marques de Mello, Faradiba Sarquis Serpa, Joseane Chiabai, Fátima Rodrigues Fernandes, Herberto José Chong-Neto, Emanuel Sávio Cavalcanti Sarinho, Norma de Paula Motta Rubini, Dirceu Solé

**Affiliations:** ^1^Department of Social Medicine, Ribeirão Preto Faculty of Medicine, University of São Paulo, São Paulo, Brazil; ^2^Brazilian Association of Allergy and Immunology (ASBAI), São Paulo, Brazil; ^3^Department of Internal Medicine, School of Sciences of Santa Casa de Misericórdia de Vitória, Vitória, Brazil; ^4^Department of Pediatrics, Federal University of Espírito Santo, Vitória, Brazil; ^5^Scientific Brazilian Association of Allergy and Immunology (ASBAI), São Paulo, Brazil; ^6^Division of Allergy and Immunology Hospital do Servidor Público Estadual de São Paulo, São Paulo, Brazil; ^7^PENSI Research Institute, São Paulo, Brazil; ^8^Department of Pediatrics, Federal University of Paraná, Curitiba, Brazil; ^9^Department of Pediatrics, Federal University of Pernambuco, Recife, Brazil; ^10^Full School of Medicine and Surgery of the Federal University of the State of Rio de Janeiro, Rio de Janeiro, Brazil; ^11^Division of Allergy and Clinical Immunology, Department of Pediatrics, Federal University of São Paulo, São Paulo, Brazil

**Keywords:** allergy and immunology, integral health care, professional profile, health policies, telemedicine

## Abstract

**Objective:**

To assess the profile of allergist/immunologist (A/I) physicians in Brazil, the workplace, the access to diagnostic and therapeutic procedures, and the impact of the COVID-19 pandemic on professional practice.

**Methods:**

This cross-sectional study was conducted as an *online* survey. All adhering members of the Brazilian Association of Allergy and Immunology (ASBAI) received a *Google Forms* tool by email. The questionnaire addressed sociodemographic and professional aspects of the Brazilian allergists/immunologists (A/I) daily routine. The information was analyzed by SPSS version 20.0.

**Results:**

Four hundred and sixty members answered the questionnaire. Women were predominant among the responders (336; 73%), and the median age was 47 years (range, 27–82 years). Most participants worked in the private sector (437, 95%), whereas 256 (47%) worked in the public sector. Among the public sector employees, 210 (82%) reported having access to some diagnostic test for allergic diseases and inborn errors of immunity. Only 91 (35%) A/I physicians in the public system had access to allergen-specific immunotherapy, compared to 416 (95, 9%) of those in the private sector. Regarding biological drugs, 135 (52.7%) and 314 (71.9%) of the A/I physicians working in the public and private sector, respectively, reported access. Two hundred and eighty-three (61.6%) had at least a 50% reduction in the number of consultations, and 245 (56%) provided telemedicine care during the COVID-19 pandemic.

**Conclusion:**

Brazilian A/I have incorporated the most recent advances in managing immunoallergic diseases into their clinical practice, but they still have little access to various diagnostic methods. Strategies to enable the presence of A/I in public health services should be discussed and implemented. The coronavirus pandemic has accelerated the incorporation of telemedicine as a viable and promising method of medical care and can expand access to the specialty.

## Introduction

In recent decades, the increased prevalence of immunoallergic diseases has driven a greater demand for qualified medical specialists working in both the private and public health services and at different levels of health care to meet the needs of the population suffering from immunological diseases (1). Simultaneously, the advances in diagnostic procedures and the development of new treatments have created the need for the continuous training of these specialists. Thus, the specialty society must work together with different spheres of the health scenario in Brazil to improve access to diagnosis and treatment of allergic and immunological conditions that affect approximately 30% of the population (1, 2).

The Brazilian Association of Allergy and Immunology (ASBAI) mission is to promote and disseminate knowledge regarding allergy and clinical immunology to strengthen the practice of the specialty with excellence, both in the public and private health sectors (2). Therefore, the ASBAI is interested in accompanying the trajectory of the Brazilian specialists in different scenarios of action to identify barriers and promote policies that guarantee better working conditions and comprehensive health care to patients with allergic diseases and inborn immune errors (IEI) (3), thus creating a stimulus for sister societies in other countries to move in a similar direction.

In a previous survey, the ASBAI evaluated the performance of its members and obtained a general panorama regarding their practicing headquarters, availability of diagnostic tests, and immunotherapy (4). In that study, the allergists/immunologists (A/I) were mainly young, and their distribution was concentrated in large centers. Access to specialized clinical allergy and immunology care was restricted to a few services, generally in colleges, making it difficult to provide comprehensive care for patients with immunoallergic diseases, especially to the ≥ 70% of the population that depends on care provided by the public system, the *Sistema Único de Saúde* (SUS). Access to diagnostic tests and specialists trained in allergen-specific immunotherapy (ASI) were also identified as shortcomings (4).

After 5 years (4), the need to update the information about the performance of A/I physicians in Brazil arose, especially during the pandemic. COVID-19 was declared a pandemic in March 2020 (5, 6). It impacted medical care and imposed several challenges on A/I. They were required to follow social distancing recommendations without abandoning their responsibility of providing quality care for their patients.

The objective of this study was to verify the current situation of the A/I in Brazil regarding their workplace, access to diagnostic resources, treatments, and the impact of the COVID-19 pandemic on their daily clinical practice.

## Methods

The study comprised an *online*, cross-sectional survey conducted from March to May 2021. All current ASBAI members were invited to participate. They were sent e-mails containing information about the research and links to *Google Forms®* and the email address to access the questionnaire (questionarioasbai@gmail.com) weekly.

The questionnaire addressed sociodemographic and professional aspects in 34 multiple-choice questions and one open-answer question (questionnaire in Supplementary Material). The collected information was electronically and automatically transferred from *Google Forms* to a Microsoft Excel® spreadsheet. At the end of the collection period, the database was transferred to SPSS version 20.0. Data were checked for duplicity and consistency and cataloged as continuous variables (age in years) and categorical variables (the others). The data were analyzed, presenting the results as medians and absolute and relative frequency distributions (proportions).

## Results

**Figure 1:**
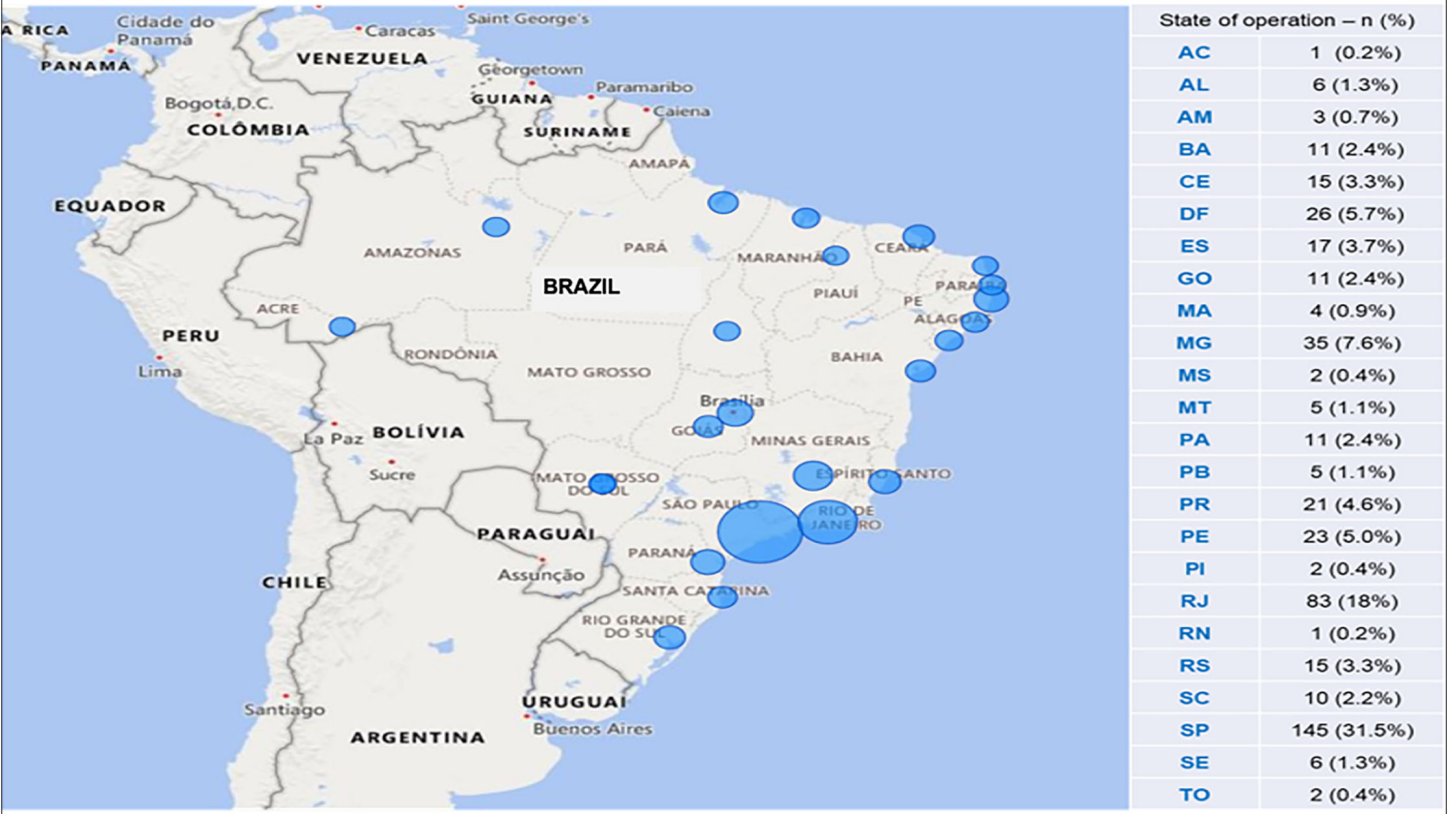
Distribution of participants, according to the state of residence. AC, Acre; AL, Alagoas; AM, Amazonas; BA, Bahia; CE, Ceará; DF, Distrito Federal; ES, Espírito Santo; GO, Goiás; MA, Maranhão; MG, Minas Gerais; MS, Mato Grosso do Sul; MT, Mato Grosso; PA, Pará; PB, Paraíba; PR, ‘Paraná; PE, Pernambuco; PI, Piauí; RJ, Rio de Janeiro; RN, Rio Grande do Norte; RS, Rio Grande do Sul; SC, Santa Catarina; SP, São Paulo; SE, Sergipe; TO, Tocantins.

*The online* questionnaire was entirely answered by 460 members, corresponding to 24.9% of the current ASBAI members (*n* = 1,848). A total of 134 questionnaires were rejected due to duplicity or incompleteness. The distribution of these specialists throughout Brazil was heterogeneous, with a predominance in the southeastern region, especially in state capitals. It remained proportionally similar to the observed distribution of ASBAI members in 2021 (Figure 1).

Regarding the demographic profile, women were predominant (336, 73%) in all the age groups. The ages ranged from 27 to 82 years (median, 47 years).

Most physicians practiced only in allergy and immunology, whereas 36.3% worked as pediatricians. Despite this, few restricted their care to pediatric patients. Less than half of them worked in the public health services as specialists, and most of them worked in the private health services (Table 1).

**Table 1:** The primary specialty, age group of the patients, and workplace of Brazilian allergists/immunologists.

**Feature**		**Total** ***n*** = **460 (%)**
***The primary specialty***		
Allergy and Immunology		449 (97.6)
Allergy, Immunology, and Pediatrics		169 (36.7)
***The age group of patients treated***		
All age groups		373 (81.1)
Children and teenagers		67 (14.6)
***Do you attend to allergic diseases in the public health service?***		
Yes		256 (55.7)
***Do you work in the private health sector?***		
Yes		437 (95)
***Place of work (health services)***	**Public−256 (%)**	**Private−437 (%)**
Basic health unit	27 (10.5)	–
General hospital outpatient clinic	46 (18)	–
University hospital outpatient clinic	127 (49.6)	–
Office	–	392 (89.7)
Multi-specialty clinic	–	96 (22)
Private hospital outpatient clinic	–	119 (27.2)
Supplementary health outpatient clinic	–	30 (6.9)
Others	96 (37.5)	–
***Do you attend patients having or suspected of inborn errors of immunity?***		
	201 (78.5)	–
***Do you attend hospitalized patients with allergic diseases?***		
	139 (54.3)	232 (53.1)
***Do you treat hospitalized patients with inborn errors of immunity?***		
	120 (46.9)	269 (61.6)

Among the specialists working in public health services, their primary workplace was university hospitals' outpatient clinics. Among those who worked in the private health services, most did so in private practice, and one-third worked in outpatient clinics of private hospitals (Table 2). In the public sector, most specialists attended to patients with a diagnosis or suspicion of inborn errors of immunity (IEI), and half of them listened to patients hospitalized for allergic diseases or IEI. In the private sector, half of the A/I attended to patients hospitalized for allergic diseases, and two-thirds attended to patients hospitalized for IEI (Table 2).

**Table 2:** Distribution of specialists according to the availability of subsidiary exams employed in the evaluation of patients with immunoallergic diseases, in the public health services (*n* = 256).

**Laboratory research**	***n*** = **256 (%)**
***Access to allergy diagnostic tests***
Yes	210 (82)
***What tests do you have access to?***
Skin prick tests	135 (52.7)
Patch tests	92 (35.9)
Total serum IgE dosage	226 (88.3)
Specific serum IgE dosage	163 (63.7)
Oral food provocation test	116 (45.3)
Oral drug provocation test	103 (40.2)
No	23 (9)
***Access to diagnostic tests for inborn errors of immunity?***
Yes	205 (80.1)
***What tests do you have access to?***
Serum immunoglobulins (G, A, M and E)	225 (87.9)
IgG subclasses	94 (36.7)
Vaccine antigen antibodies (rubella, polio, other)	166 (64.8)
Antibodies to polysaccharides (pneumococci)	55 (21.5)
Delayed skin tests	68 (26.3)
Immunophenotyping of T lymphocytes (CD4, CD8)	155 (60.5)
Immunophenotyping of B lymphocytes (CD16, CD20)	111 (43.4)
Immunophenotyping of NK lymphocytes (CD56)	87 (34)
Phagocyte evaluation (dihydro-rodamine)	23 (9)
Complement and fractions	165 (64.4)
C1 inhibitor qualitative and quantitative	73 (28.5)
Newborn screening—TRECs/KRECs	23 (9)
Other	20 (7.8)

Most specialists in the public health services had access to diagnostic tests for allergic diseases (Table 3). The most available were the total serum Immunoglobulin E (IgE), serum antigen-specific IgE, and skin tests for immediate hypersensitivity (Table 3). Less than half of the specialists had access to provocation tests with food or drugs. Regarding patients with suspected IEI, most specialists had access to quantifying tests for serum immunoglobulins, anti-vaccine antigen antibodies, immunophenotyping and counting of T lymphocytes, and a total complement and fractions, among others (Table 3).

**Table 3:** Access to allergen-specific immunotherapy and biologics for patients in public and private health services, according to the specialists' responses—*n* (%).

**Variable**	**Public**−**256 (%)**	**Private**−**437 (%)**
***Access to immunotherapy***		
Yes	91 (35.5)	419 (95.9)
***Access to biologics***		
Yes	135 (52.7)	314 (71.9)
***Prescription of biologics***		
Yes	157 (61.3)	–
***Diseases biologics were prescribed for***		
Asthma	88 (34.4)	188 (40.9%)
Urticaria	119 (46.5)	259 (56.3%)
Atopic dermatitis	74 (28.9)	195 (42.4%)
IEI	97 (37.9)	52 (11.3%)
***Available immunobiological***		
Omalizumab	142 (55.5)	265 (60.6)
Dupilumab	66 (25.8)	122 (27.9)
Mepolizumab	14 (5.5)	24 (5.5)
Benralizumab	–	23 (5.3)
Human Immunoglobulin	121 (47.3)	170 (38.9)
Other	31 (12.1)	13 (3)
No	43 (16.8)	128 (29.3)
In private health services, how do you access immunobiological?^*^		
SUS—Unified health system	–	82 (25.9)
Via health insurance company	–	245 (77.5)
Judicialization	–	253 (80.1)
Own resource	–	58 (18.4)
I have no patient in use	–	121 (38.3)

* *Considering only the prescribers (n = 316)*.

Only one-third of the specialists in the public health services reported having access to ITA, compared to almost all of those working in the private health services. Furthermore, half and two-thirds of the specialists working in the public and private sectors have access to biological agents. Among the specialists working in the public sector, slightly more than half reported prescribing at least one of these agents (Supplementary Table 1).

As for the diseases for which biological drugs were prescribed by professionals working in the public health services, urticaria, IEI, asthma, and atopic dermatitis were notable in descending order. The main prescribing indications in private health services were chronic spontaneous urticaria, atopic dermatitis, asthma, and IEI (Supplementary Table 1).

Omalizumab and dupilumab were the most used biological agents in public and private health services (Supplementary Table 1). Half of the specialists in the public sector and one-third in the private sector reported using it. Regarding patient access to biological agents in the private sector, judicialization, and health insurance companies were the most frequently used access routes (Supplementary Table 1).

Regarding the impact of the pandemic on private care, more than half of the A/I participating in the survey reported a reduction in the number of consultations by at least 25% and reported providing support by telemedicine to their patients.

**Figure 2:**
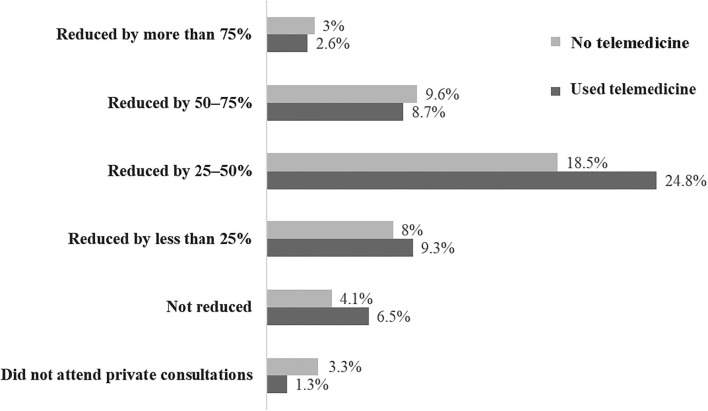
Distribution of participants (*n* = 460) according to the reduction in consultations in a private office and the option for telemedicine care.

Another relevant finding was that approximately half of the specialists who answered the questionnaire work in the public health services, and 29.5% of those who do not would like to do so (Figure 2).

## Discussion

Although the initial records on allergy specialty date back to the beginning of the twententh century, its structuring as a medical specialty in Brazil has been more recent, having only been established for 50 years (2).

Unlike some parts of the world, allergy and immunology is a recognized medical specialty in Brazil and confers the title of specialist. In Europe, a study conducted by the *European Academy of Allergy and Clinical Immunology* (EAACI) revealed that allergy is not recognized as a medical specialty or subspecialty in some countries (Austria, Belgium, Denmark, Slovenia, and the Republic of Ireland) and is recognized as a subspecialty in others (Germany, Finland, the Netherlands, Hungary, and Turkey) (7).

Despite the recognition of allergy as a medical specialty, the distribution of Brazilian A/I, which comprised a higher proportion of female and young physicians, was heterogeneous and more concentrated in the southeastern region, particularly in the states of São Paulo, Rio de Janeiro, and Minas Gerais, similar to what was observed in a previous^3^ study. Our findings agree with the Scheffer et al. study from the 2020^8^ Brazilian Medical Demography Survey. They identified the existence of 1,903 specialists in allergy and immunology in the year of the survey, representing.4% of the total number of physicians with specialization in the country (n = 47,575) or.91 specialists per 100,000 inhabitants, thereby highlighting the scarcity of these professionals to attend patients with allergic and immunologic diseases. They also reported their heterogeneous distribution throughout the country and their concentration in large urban centers (8).

The completion rate of the structured questionnaire was 100%, indicating that all physicians who started the survey completed it. We achieved a response rate of 25% within the expected range for the research strategy adopted. Furthermore, the sample studied adequately represented the target population, which minimized the risk of non-response bias and ensured the reliability of the results (9). Moreover, the response rate in our study was 5% higher than that in a previous study (4), suggesting increased interest in issues related to the specialty by ASBAI members. The present study showed that most participants work exclusively as A/I, mainly in private health services, and assist patients of all ages. Although the A/I predominantly work in the private health services, more than half of them reported working also in the public health service, which indicates a trend toward more significant equity in access to specialized care. However, investigating the main places of work of specialists in public health services, we found that almost all A/Is work in outpatient clinics of university hospitals, which means that specialists are concentrated in the more complex services of the SUS and large urban centers. As these are services with regulated access and the flow depends on a local-regional regulatory system, the patient cannot access them directly. So, to succeed in reaching an A/I expert, the patient needs to be inserted into a wellorganized health care network, where all service points (primary care units, specialized care units, high-complexity hospitals, and service providers such as diagnostic service units) act in a coordinated way, and the regulatory system works appropriately. However, all those aspects ultimately depend on the local management of the SUS. The difficulties and obstacles faced by patients with severe allergic diseases and IEI within regulatory networks can result in delays in diagnosis, irreversible health damage, and deaths due to late initiation of treatment. Therefore, it is necessary to encourage this discussion with local managers to bring proposals that guarantee the improvement of flows and access to our specialty, especially for the population residing in cities geographically distant from teaching hospitals.

On assessing the type of assistance, we found that those working in the private health services as those working in the SUS provide care for patients hospitalized for allergic diseases and IEI. These findings suggest the complex level of training A/Is should have for full professional practice. In this sense, permanent and continuous education strategies are crucial to ensure high quality of health care for patients with immunoallergic diseases.

However, the quality of health care to patients did not depend only on the specialist's skills and competencies but also on the diagnostic and therapeutic resources available in the different sectors, whether public or private. The present study identified that complementary tests, especially laboratory tests for diagnosing both allergic diseases and IEI and are available to most A/I in public and private services. Regarding the “*in vivo”* tests, the skin prick tests are the most accessible to specialists in the SUS. In contrast, the least available are the contact tests, compromising the proper approach to suspected contact dermatitis. In the previous study, 45.6% of the A/I participants reported having access to epicutaneous tests (4).

Similarly, tests to assess the antibody-mediated response and complement system were the most frequently available for investigating suspected IEI in the current study, in agreement with the data from the previous study (4). However, tests to investigate the cellular and phagocytic responses are less available, leading to delays in diagnosing and treating severe immune deficiencies.

Regarding allergen-specific immunotherapy (ASI), its availability in SUS is reported to be three times lower than that in private services, suggesting inequity of access. It is critical to the management of some allergic diseases because it is the only therapeutic tool that modulates the immune system, inducing tolerance and preventing the development of new sensitizations. It requires specific training and should be adequately proposed for its medical use in an individualized and safe manner, justifying the difference observed. The difficulty in obtaining allergenic extracts can also explain the problem of implementing ASI in public health services. There is no pressure from the industry as that for anti-infection vaccines. As a result, patients do not benefit from the ASI, and the inadequate control of the allergic diseases leads to increased costs.

Regarding biological agents, even though they are recent and more expensive options, more than half of the A/Is reported having prescribed them for patients with allergic diseases and IEI in public and private sectors. This suggests that Brazilian professionals are aware of the rapid changes in clinical protocols and new therapeutic options for their patients. Omalizumab, which was approved by the National Health Surveillance Agency (ANVISA) more than 10 years ago for severe asthma and, later, for chronic spontaneous urticaria (CSU); dupilumab, approved more recently for atopic dermatitis, asthma, and chronic rhinosinusitis (CRS) with polyps; and, intravenous human immunoglobulin for IEI, is the most commonly mentioned. Immunobiological are generally expensive, and only a few patients for whom these agents are indicated can use them. Thus, to use these products, most patients need subsidized access. The judicialization and costing by health insurance companies are the forms of access most frequently reported by the research participants.

The Clinical Protocol and Therapeutic Guideline (CPTG) for asthma has been recently updated and included two biological agents for severe asthma—mepolizumab and omalizumab. In addition, the National Health Agency (ANVISA) updated the Roll of Procedures and Health Events, making it mandatory for private health care plans to cover a series of procedures, including immunobiological therapy with benralizumab, mepolizumab, and omalizumab for severe asthma and omalizumab for chronic spontaneous urticaria, provided that they meet the criteria established by the Guidelines for Use (10). Access to effective and safe therapies to treat severe forms of allergic and immunological diseases is a significant achievement in ensuring good quality of life for patients (10).

The COVID-19 pandemic also significantly affected the A/I routine. More than half of the specialists reported a decrease in medical appointments by at least 50%. However, more than half of the affected professionals reported using telemedicine as a resource to reduce the negative financial impact and maintain continually specialized care for patients, complying with the recommendations of national and international health authorities for social isolation (11). The pandemic seems to have consolidated the essential role of connectivity in several sectors. In medicine, telemedicine appointments might become a necessary option for providing health care, in line with the situation in other places worldwide (12–16).

Currently, the ASBAI has offered its members alternatives to ensure continuous education with updated and relevant content. The *online* model has enabled greater access to information and should impact the daily practice of the specialist. The ASBAI University (www.universidade.asbai.org.br) is the platform where all the produced digital content is stored and made available free of charge to the A/I physician. There was a 20-fold increase in enrollments in its courses during the pandemic, thereby consolidating distance learning to update the specialist.

In conclusion, the Brazilian A/I have followed the fast growth of the specialty and tried to incorporate the new therapies into clinical practice. The access to tests has increased, but food and drug allergies investigations depend on oral provocation tests (OPT). OPT and other tests, such as those for investigating IEI, also need health policies to enable their incorporation into services. In addition, governmental authorities must be aware of the need to enlarge health care for patients with allergic diseases and IEI in the SUS. Implementing specialized health care services in allergy and immunology all over the country, especially in those regions with a lack of specialists, establishing lines of care, reference/counter-reference flow guarantees access to quality care for patients. The COVID-19 pandemic impacted the entire health sector, affecting specialty practice, especially during the restricted in-person care. However, the specialty has benefited from the discussion and anticipated incorporation of telemedicine into the routines of specialists in allergy and immunology.

## Data availability statement

The raw data supporting the conclusions of this article will be made available by the authors, without undue reservation.

## Ethics statement

The studies involving human participants were reviewed and approved by Brazilian Association of Allergy and Immunology Ethics Committee. The patients/participants provided their written informed consent to participate in this study.

## Author contributions

LM, FS, JC, FF, HC-N, ES, NR, and DS: preparation of the questionnaire and writing and reviewing the manuscript. All authors contributed to the article and approved the submitted version.

## Conflict of interest

The authors declare that the research was conducted in the absence of any commercial or financial relationships that could be construed as a potential conflict of interest.

## Publisher's note

All claims expressed in this article are solely those of the authors and do not necessarily represent those of their affiliated organizations, or those of the publisher, the editors and the reviewers. Any product that may be evaluated in this article, or claim that may be made by its manufacturer, is not guaranteed or endorsed by the publisher.

## Abbreviations

ANVISA: National Health Surveillance Agency
ASBAI: Brazilian Association of Allergy and Immunology
A/I: Allergists and Immunologists
COVID-19: Coronavirus disease 2019
CSU: chronic spontaneous urticaria
CRS: chronic rhinosinusitis
IEI: inborn immune errors
ASI: allergen-specific immunotherapy
CPTG: Clinical Protocol and Therapeutic Guideline
SUS: Sistema Único de Saúde.
