# Supplementary material for: The specialty of allergy and clinical immunology in Brazil
Source: Front Allergy. 2022 Jul 18;3:933816. doi: 10.3389/falgy.2022.933816 (PMC9347215; doi:10.3389/falgy.2022.933816)
Supplement: Supplementary file 1 [file Data_Sheet_1.docx]

| \| 1.Sex: \| Male  Female \| \| --- \| --- \| \| 2. Age (complete years): \|  \| \| 3. City of residence: \|  \| \| 4. State of residence: \|  \| \| 5. Main acting specialty (you can tick more than one option): \| Allergy / Immunology  Pediatrics  Medical clinic  Family Health  Other: \| \| 6. What is the age range of your patients considering the area of expertise Allergy/Immunology? \| Children and teenagers  All age groups  Teenagers and adults  Adults \| \| 7. Acts as an allergist in the public service? \| No  Yes \| \| 8. Where do you work as an allergist in the public service? (tick as many as needed) \| Basic health Unit  General Hospital Outpatient Clinic  Outpatient University Hospital (teaching)  Other \| \| 9. You see patients with allergic diseases in the public service? \| No  Yes \| \| 10. Do you care for patients with or suspected immunodeficiencies in the public service? \| No  Yes \| \| 11. In which location do you care for patients with immunodeficiencies (innate immunity errors -EII) or suspected in the public service? \| Basic health Unit  General Hospital Outpatient Clinic  Outpatient University Hospital (teaching)  Other \| \| 12. Do you care for patients hospitalized for allergic diseases in the public service? \| No  Yes \| \| 13. Do you care for hospitalized patients with allergic diseases in the private service? \| No  Yes \| \| 14. Do you care for hospitalized patients with immunodeficiencies (IEI) in the public service? \| No  Yes \| \| 15. Do you care for hospitalized patients with immunodeficiencies (IEI) in the private service? \| No  Yes \| \| 16. Do you have access to diagnostic tests for allergy in the public service in which you work? \| No  Yes  I don't work in the public service \| \| 17. What diagnostic tests for allergy do you have access to in the public service? (tick as many as needed) \| Skin prick tests  Contact tests  Total IgE dosage  Specific serum IgE dosage  Oral challenge test with food  Oral challenge test with drugs  None  I don't work in the public service \| \| 18. Do you have access to diagnostic tests for immunodeficiencies (EII) in the public service in which you work? \| No  Yes  I don't work in the public service \| \| 19. Which diagnostic tests for immunodeficiency (EII) are available in the public service? (tick as many as needed) \| Dosage of serum immunoglobulins (G, A, M and E)  IgG subclass dosage  Antibodies to vaccine antigens (rubella, polio, among others)  Antibodies to polysaccharide antigens (pneumococci)  Late reading skin tests  Immunophenotyping and quantification of T lymphocytes (CD4, CD8)  Immunophenotyping and quantification of B lymphocytes (CD19, CD20)  Immunophenotyping of NK lymphocytes (CD56)  Evaluation of phagocytes (Rhodamine)  Complement and fractions  Quantitative and qualitative C1 inhibitor  Newborn screening - TRECs/KRECs  Others  I don't work in the public service \| \| 20. Do you have access to immunotherapy in the public service? \| No  Yes  I don't work in the public service \| \| 21. Do you have access to immunobiologicals for the treatment of immunoallergic diseases for public service patients? \| No  Yes  I don't work in the public service \| \| 22. Have you ever prescribed immunobiologicals for the treatment of immunoallergic diseases to public service patients? \| No  Yes  I don't work in the public service \| \| 23. For which disease have you already prescribed immunobiological in the public service? (tick as many as needed) \| Asthma  Urticaria  atopic dermatitis  chronic rhinosinusitis  Primary immunodeficiency (EII)  None \| \| 24. Which immunobiological do you have access to for the patients you see in the public service? (tick as many as needed) \| omalizumab  dupilumab  mepolizumab  benralizumab  human immunoglobulin  Others  I do not attend the public service  None \| \| 25. Do you work in the private sector? * \| No  Yes \| \| 26. Where do you work in the private sector? (tick as many as needed) * \| Clinic  multispecialty clinic  private hospital  Supplementary health service outpatient clinic  I don't work in the private sector \| \| 27. Do you prescribe immunotherapy in the private sector? * \| No  Yes  I don't work in the private service \| \| 28. Do you have access to immunobiologicals for private sector patients? \| No  Yes  I do not work in the private service  I have no patients in use \| \| 29. For which disease have you already prescribed immunobiological in the private service? (tick as many as needed) \| Asthma  atopic dermatitis  Urticaria  Chronic Rhinosinusitis  Primary immunodeficiency (EII)  other indications \| \| 30. How does the patient who attends in the private service have access to treatment with immunobiologicals? (tick as many as needed) \| Via health operator  own resource  Via the Unified Health System  Judicialization  I have no patients in use \| \| 31. Which immunobiological do you have access to by health operators? (tick as many as needed) \| omalizumab  dupilumab  mepolizumab  benralizumab  human immunoglobulin  Others  None \| \| 32. The pandemic reduced the number of consultations in the private office by what percentage? \| did not reduce  Reduced below 25%  Reduced between 25 and 50%  Reduced between 50 and 75%  Reduced above 75%  I don't answer in private \| \| 33. Are you attending by Telemedicine? \| Yes  No \| \| 34. If you had the opportunity, would you like to work in the Public Health System?* \| No  Yes  I don't have an opinionJá trabalho no SUS \| \| 35. Leave your comment here: \|  \| |
| --- | --- | --- | --- | --- | --- | --- | --- | --- | --- | --- | --- | --- | --- | --- | --- | --- | --- | --- | --- | --- | --- | --- | --- | --- | --- | --- | --- | --- | --- | --- | --- | --- | --- | --- | --- | --- | --- | --- | --- | --- | --- | --- | --- | --- | --- | --- | --- | --- | --- | --- | --- | --- | --- | --- | --- | --- | --- | --- | --- | --- | --- | --- | --- | --- | --- | --- | --- | --- | --- | --- |

Table 4 – Questionnaire on the specialty of Allergy and Immunology at different levels of health care
